# Functional characterization of two survival factor 1 genes in *Mucor lusitanicus*

**DOI:** 10.1128/spectrum.01103-24

**Published:** 2024-08-27

**Authors:** Olivér Jáger, Csilla Szebenyi, Tammam Khaliefeh Siliman Abu Saleem, Anna Molnár, Vanda Kovács, Karina Kiss, Mónika Homa, Bernadett Vágó, Sándor Kiss-Vetráb, Mónika Varga, Rita Sinka, Csaba Vágvölgyi, Gábor Nagy, Tamás Papp

**Affiliations:** 1 Department of Microbiology, Faculty of Science and Informatics, University of Szeged, Szeged, Hungary; 2 HUN-REN-SZTE Fungal Pathomechanisms Research Group, University of Szeged, Szeged, Hungary; 3 Department of Genetics, Faculty of Science and Informatics, University of Szeged, Szeged, Hungary; 4 University of Szeged, Centre of Excellence for Interdisciplinary Research, Development and Innovation (SZTE IKIKK), Fungal Pathomechanisms Research Group, Szeged, Hungary; University of Debrecen, Debrecen, Hungary

**Keywords:** mucormycosis, key regulator, cold stress, sphingolipid biosynthesis, pathogenicity, CRISPR-Cas9

## Abstract

Survival factor 1 (Svf1) protein has been described in some ascomycetous fungi where it was found to be contributing to several essential physiological processes, such as response to osmotic, oxidative and cold stresses, sphingolipid biosynthesis, morphogenesis, sporulation, antifungal resistance, and pathogenicity. It was also suggested that it can be a novel central regulator affecting the expression of various genes. In the present study, function of this protein and the encoding genes is described for the first time in a fungus (i.e., in *Mucor lusitanicus*) belonging to the order Mucorales. *M. lusitanicus* has two putative *svf1* genes named *svf1a* and *svf1b*. Expression of both genes was proven. Although the expression of *svf1a* was affected by several environmental stresses and knocking out the gene affected adaptation to low temperatures and the sporulation ability, the main survival factor functions, such as participation in the maintenance of the viability, the response to oxidative and cold stresses, and the sphingolipid biosynthesis, could be associated with Svf1b, suggesting a central regulatory role to this protein. Interestingly, knockout of both genes affected the pathogenicity of the fungus in a *Drosophila* model.

**IMPORTANCE:**

*Mucor lusitanicus* is a widely used model organism to study various biological processes in the basal fungal group Mucorales. Several members of this group can be agents of mucormycosis, an opportunistic fungal infection, which is associated with high mortality, rapid progression, and wide resistance to the commonly used antifungal agents. Svf1 proteins have so far only been identified in fungi, where they have been involved in pathogenicity and resistance to antifungal agents in many cases. Only a limited number of factors affecting the stress response, antifungal resistance, and virulence of Mucorales fungi have been revealed. Elucidating the function of a fungus-specific protein that may regulate these processes may bring us closer to understanding the pathogenesis of these fungi.

## INTRODUCTION

Survival factor 1 (Svf1) protein and the encoding gene were first described in *Saccharomyces cerevisiae* where it was found to be required for the diauxic growth shift and survival under stress conditions (1). Svf1 has also been characterized, at least partially, in a few fungal species, such as *Fusarium fujikuroi* (2), *Fusarium graminearum* (3), *Sclerotinia sclerotiorum* (4), and *Aspergillus nidulans* (5). In *S. cerevisiae*, it was demonstrated that Svf1 is necessary for survival under oxidative and cold stresses (6). In addition to affecting the response to osmotic, oxidative, and cold stresses, Svf1 was found to contribute to the fungicide resistance in plant pathogenic *Fusarium* species (2, 3). In *Schizosaccharomyces pombe*, mutation of the *svf1* gene caused a hypersensitivity to the antifungal agent, micafungin (7). Furthermore, transcriptomic analysis of human pathogenic *Paracoccidioides* spp. found the *svf1* gene to be differentially expressed in response to itraconazole and sulfamethoxazole treatments (8, 9). It was suggested that Svf1 affects survival by modulating the sphingolipid metabolism and regulating the sphingoid base pathway in yeast (10). Moreover, in *F. graminearum* and *A. nidulans*, the activity of Svf1 was found to be essential to the normal morphogenesis, especially to the radial growth, the asexual and sexual spore formation, and pathogenicity (3, 5, 11). As transcription analyses indicated that it affects the expression of development regulatory genes, Lim et al. (5) proposed that Svf1 might be a novel central regulator of growth, differentiation, and secondary metabolism.

*Mucor lusitanicus* (formerly known as *M. circinelloides* f. *lusitanicus*) is a filamentous fungus (12) and a widely used model organism to study various biological processes, such as pigment (13, 14) and biofuel production (15, 16), RNA interference (17), morphogenesis (18–20), or pathogenicity (3, 21, 22). *Mucor* species may also have biotechnological importance as enzymes (23), carotenoids (24), fatty acids, and organic acid producers (25, 26). Several Mucorales species have been reported as agents of a life-threatening opportunistic fungal infection known as mucormycosis (27, 28). Such infections, which are notorious for their rapid progression, high mortality rates, and difficult diagnosis and treatment (27, 29), are most frequently associated with an immunocompromised state and diabetic ketoacidosis in human patients. In the past 2 years, a conspicuously high number of mucormycosis cases have been reported among COVID-19 patients (30–32).

The genome of *M. lusitanicus* contains two Svf1-encoding genes, which were named *svf1a* and *svf1b*. The aim of the present study was to examine their expression and function, first in a Mucorales fungus.

## RESULTS

### Identification and transcription analysis of the *svf1* genes

In the *M. lusitanicus* genome (DoE Joint Genome Institute; *M. lusitanicus* CBS277.49v2.0; http://genome.jgi-psf.org/Mucci2/Mucci2.home.html), two hypothetical *svf1* genes were identified by a similarity search using the amino acid sequence of *S. cerevisiae* Svf1 [UniProt ID: Q05515; (6)] and named as *svf1a* (Protein ID: 154889) and *svf1b* (Protein ID: 166561). The amino acid identity of the putative Svf1a and Svf1b proteins to the yeast Svf1 was 26% (similarity 41%) and 33.5% (similarity 42%), respectively, and 63% (similarity 80%) compared with each other. The *svf1*-like domain [PF08622 (6)] was identified in both putative Svf1 proteins in the 53–381, and 54–379 amino acid positions for Svf1a and Svf1b, respectively.

**Fig 1:**
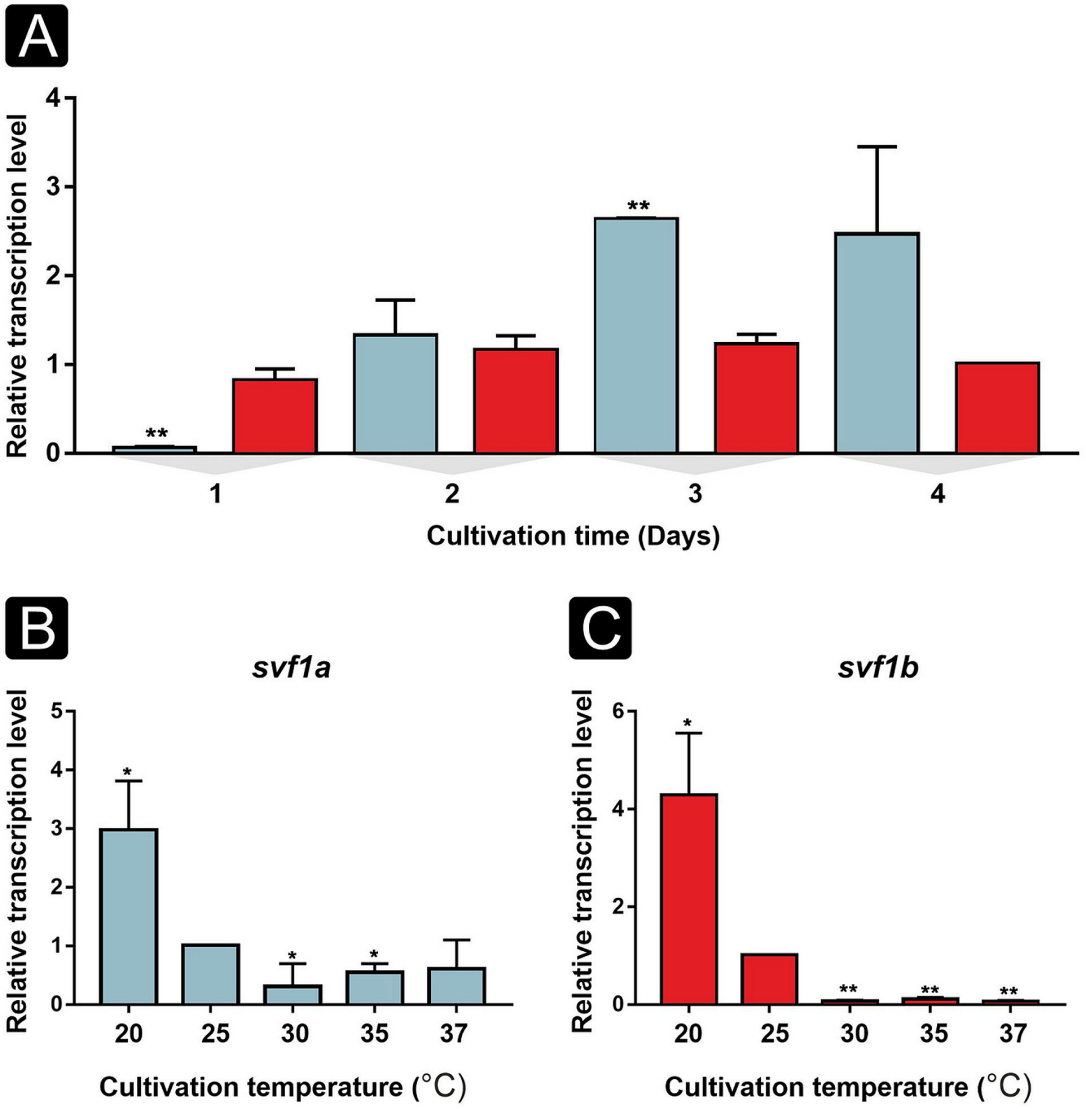
Transcription of the *M. lusitanicus svf1* genes during the cultivation of the fungus (**A**) and at different temperatures (**B and C**). (**A**) Relative transcription level of the *svf1a* (blue) and *svf1b* (red) genes of the strain MS12 grown on minimal medium at 25°C during the first 4 days of cultivation (the relative transcript level of *svf1b* at the fourth day of cultivation was taken as 1; *P* values were calculated according to the unpaired *t*-test statistical method; values indicated with asterisks significantly differed from the values taken as 1 according to **<*I>P*  ≤  0.01.). Relative transcript level of *svf1a* (**B**) and *svf1b* (**C**) genes after cultivation on minimal medium for 4 days at different temperatures (the transcript level of each gene measured at 25°C was taken as 1). Values presented are from three independent cultivations; error bars indicate standard deviation. *P* values were calculated using the two-sample, paired *t*-test statistical method. Values indicated with asterisks significantly differed from the values taken as 1 according to *P  ≤  0.05, **P  ≤  0.01.

**Fig 2:**
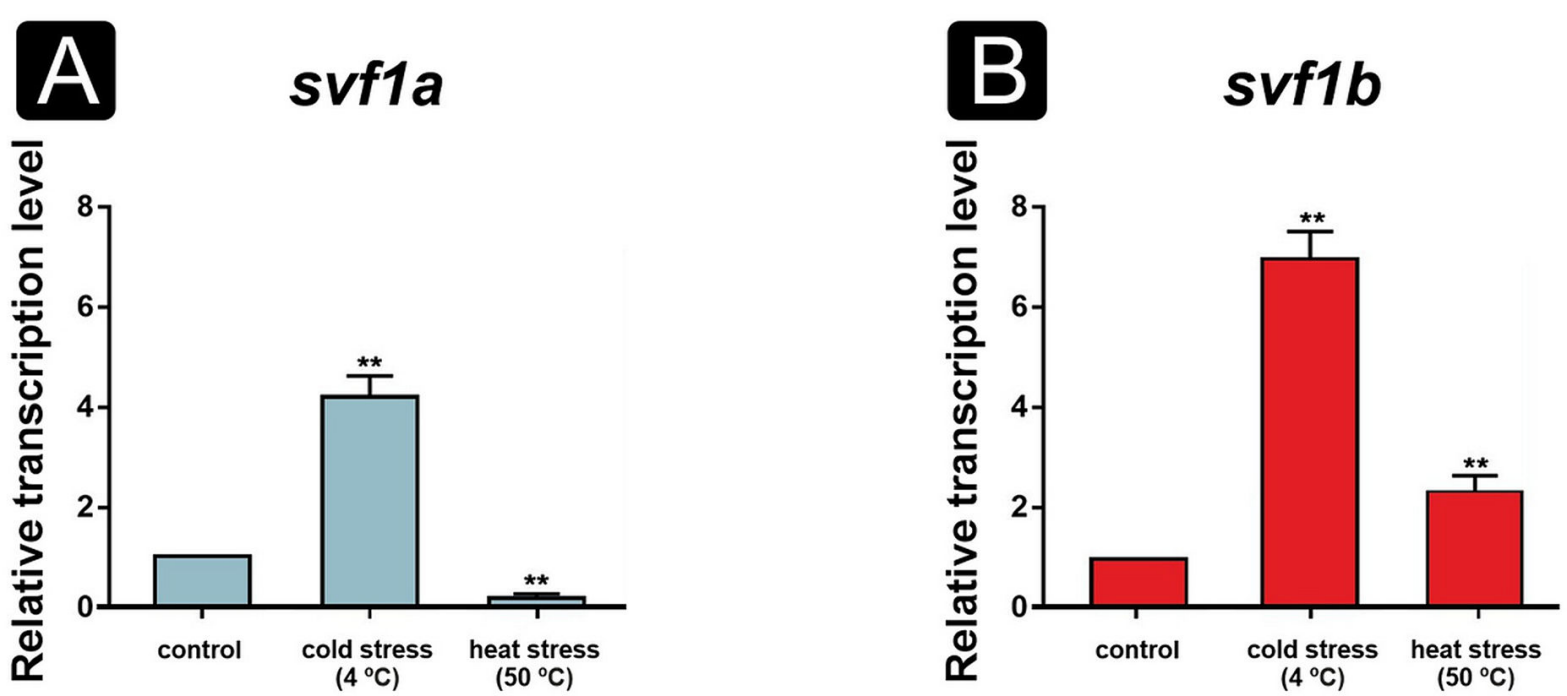
Relative transcription level of *M. lusitanicus svf1a* (**A**) and *svf1b* (**B**) after cold (4°C) and heat (50°C) stress. Transcription level of both genes measured in the mycelium of MS12 on the second day of cultivation in liquid minimal medium (YNB broth) at 25°C without stress was taken as 1. The presented values are averages of three independent experiments; error bars indicate standard deviation. Relative transcript values followed by ** significantly differed from the values taken as 1 according to the paired *t*-test (**P  ≤  0.01).

Both *svf1* genes were expressed during the cultivation (Fig. 1A). Although *svf1b* showed a constant expression throughout the cultivation period, *svf1a* reached higher transcription levels on the third day of cultivation than *svf1b* (Fig. 1A). Transcription of both genes was remarkably higher at 20°C than at elevated temperatures (Fig. 1B and C). Furthermore, *svf1* genes were found to be downregulated at temperatures higher than 25°C (Fig. 1B and C). After a cold stress (i.e., incubation at 4°C for 30 min), both *svf1a* and *svf1b* genes proved to be upregulated in the hyphae compared with the control conditions (Fig. 2A and B). After a heat shock (i.e., incubation at 50°C for 30 min), *svf1a* was found to be downregulated, whereas *svf1b* was upregulated compared with the control temperature (i.e., 25°C) (Fig. 2A and B).

**Fig 3:**
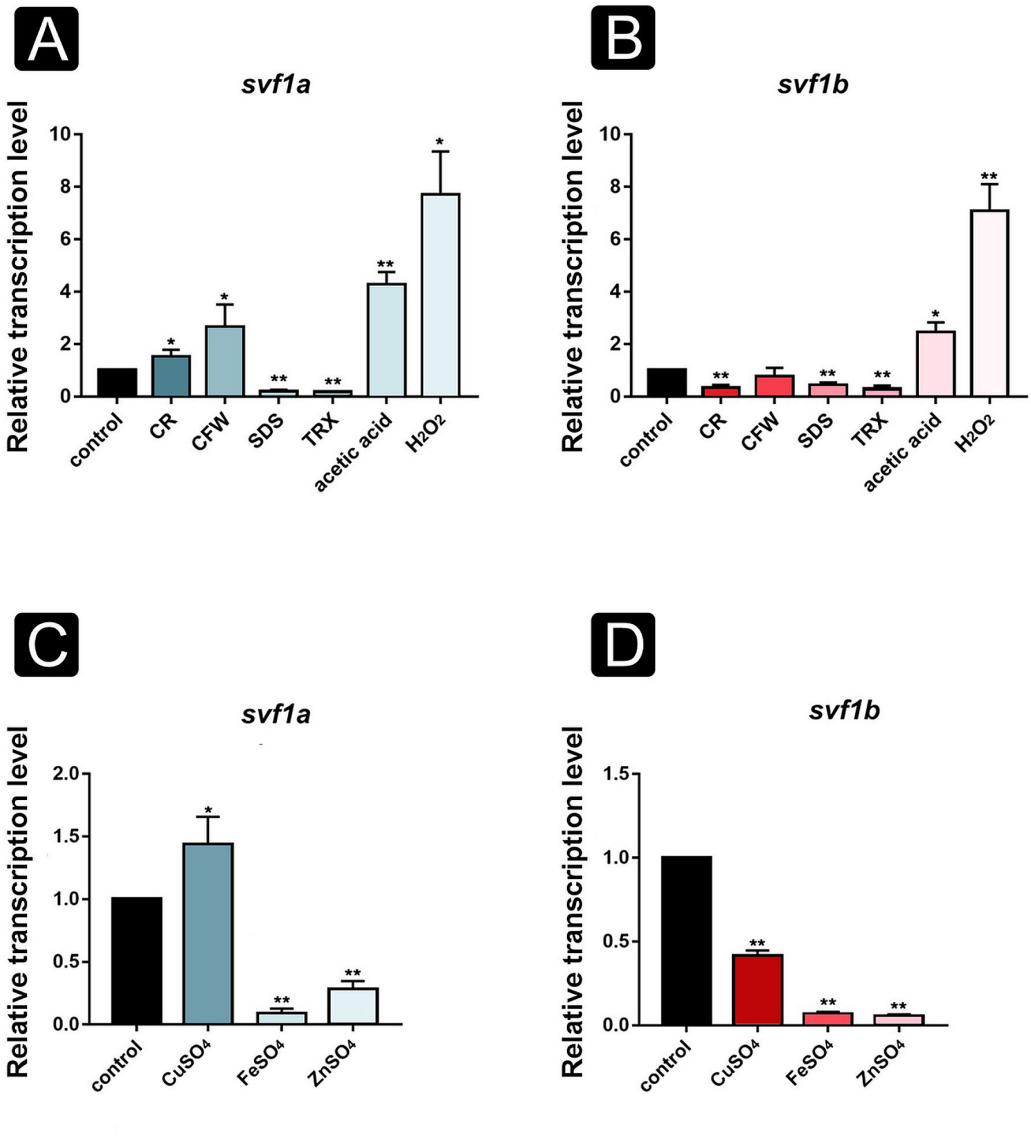
Effect of different stressors on the expression of the *M. lusitanicus svf1* genes. Relative transcription level of *svf1a* (**A**) and *svf1b* (**B**) in the presence of the stressors CR, CFW, SDS, TRX, acetic acid, and hydrogen peroxide on the second day of cultivation at 25°C. Relative transcription level of *svf1a* (**C**) and *svf1b* (**D**) genes in the presence of different heavy metal agents. In each panel, the transcription of the corresponding gene measured on YNB without any agents was taken as 1. The presented values are averages of three independent cultivations; error bars indicate standard deviation. Values followed by * or ** significantly differed from the value taken as 1 according to the paired *t*-test (*<*I>P*  ≤  0.05, **<*I>P*  ≤  0.01).

Relative transcription level of the *svf1* genes was also measured after cultivation in the presence of the cell wall stressors Congo red (CR) and calcofluor white (CFW). In the presence of CR, transcription level of *svf1a* significantly increased while that of *svf1b* decreased compared with those measured on minimal medium (Fig. 3A and B). CFW treatment also resulted in an increased expression level of *svf1a*, but it had no effect on that of *svf1b* (Fig. 3A and B). Detergents affecting the cell membrane, such as sodium-dodecyl sulphate (SDS) and Triton-X-100 (TRX), decreased the transcription level of both *svf1* genes (Fig. 3A and B).

Relative transcription level of both *svf1* genes was measured after mitochondrial and cellular oxidative stress induced by acetic acid and hydrogen peroxide, respectively. Both genes displayed elevated transcription levels in the presence of acetic acid and hydrogen peroxide (Fig. 3A and B). Heavy metals can cause chemical stress and formation of reactive oxygen species (33). To investigate the effect of heavy metals on the relative transcript level of the genes, sublethal concentrations of the tested heavy metals were used. To determine the sublethal concentrations of the heavy metals, a microdilution assay was performed (Table S1). A general decrease in the gene expression of both genes was observed in the presence of heavy metal agents (Fig. 3C and D), except for 0.5 mM CuSO_4_, which caused a significantly elevated transcription level of *svf1a* (Fig. 3C).

### *Svf1b* is required for normal vegetative growth and the viability of spores

**Fig 4:**
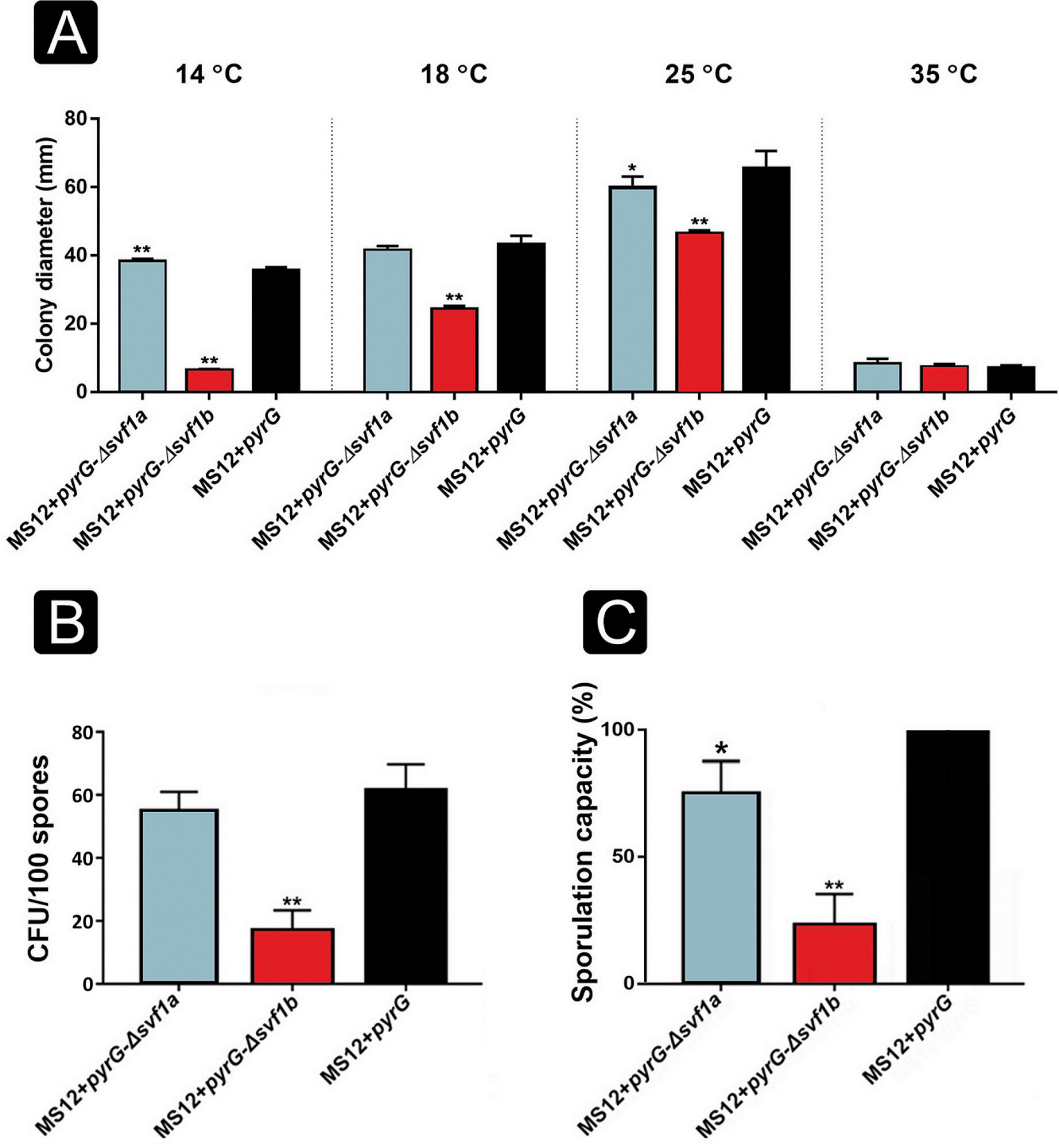
Characterization of *svf1* knockout mutants. Growth of *svf* mutants at different temperatures (14°C; 18°C; 25°C; 35°C) on the fourth day of cultivation (**A**). Viability of *svf1* mutants on minimal media (**B**). Colony forming units from 100 spores were counted on the second day of cultivation period. Spore production was evaluated by counting the number of spores produced by 4-day-old cultures (**C**). The presented values are averages of three independent experiments; error bars indicate standard deviation. Values were calculated according to the two-sample unpaired *t* test statistical method. Values indicated by asterisks are significantly different from the value of the MS12 + *pyrG* strain measured on the same day (*, *P*  ≤  0.05; **, *P*  ≤  0.01).

The *M. lusitanicus* strain MS12 *+ pyrG*-Δ*svf1b*, in which the *svf1b* gene was knocked out, exhibited significantly reduced growth at medium and lower temperatures (i.e., at 14, 18, and 25°C) compared with the control strain (MS12 + *pyrG*) (Fig. 4A). As the temperature decreased, the growth reduction of the Δ*svf1b* mutant became more pronounced. Apart from the growth intensity of these two strains, the basic hyphal morphology of the mutants did not differ from that of the original strain (Fig. S1). However, when exposed to a temperature of 14°C, we observed notable cytoplasmic releases in the Δ*svf1b* mutant, characterized by the extrusion of cytoplasmic contents. This distinct phenotype strongly implies heightened cold sensitivity, a phenomenon conspicuously absent in the control strain (Fig. S1). Moreover, colonies of the MS12 + *pyrG*-Δ*svf1b* isolates had a more intense yellow color than that of the control strain (MS12 + *pyrG*) and had an increased carotenoid content (Fig. S2). In case of the MS12 + *pyrG-Δsvf1b* mutant, the growth defect may be in relation to the significant reduction of viability of spores (Fig. 4B). Decreased spore production capacity of the *svf1b* mutant seems to be also linked with the reduced viability (Fig. 4C).

### Effect of different stressors on the growth of the *svf1* knockout mutants

**Fig 5:**
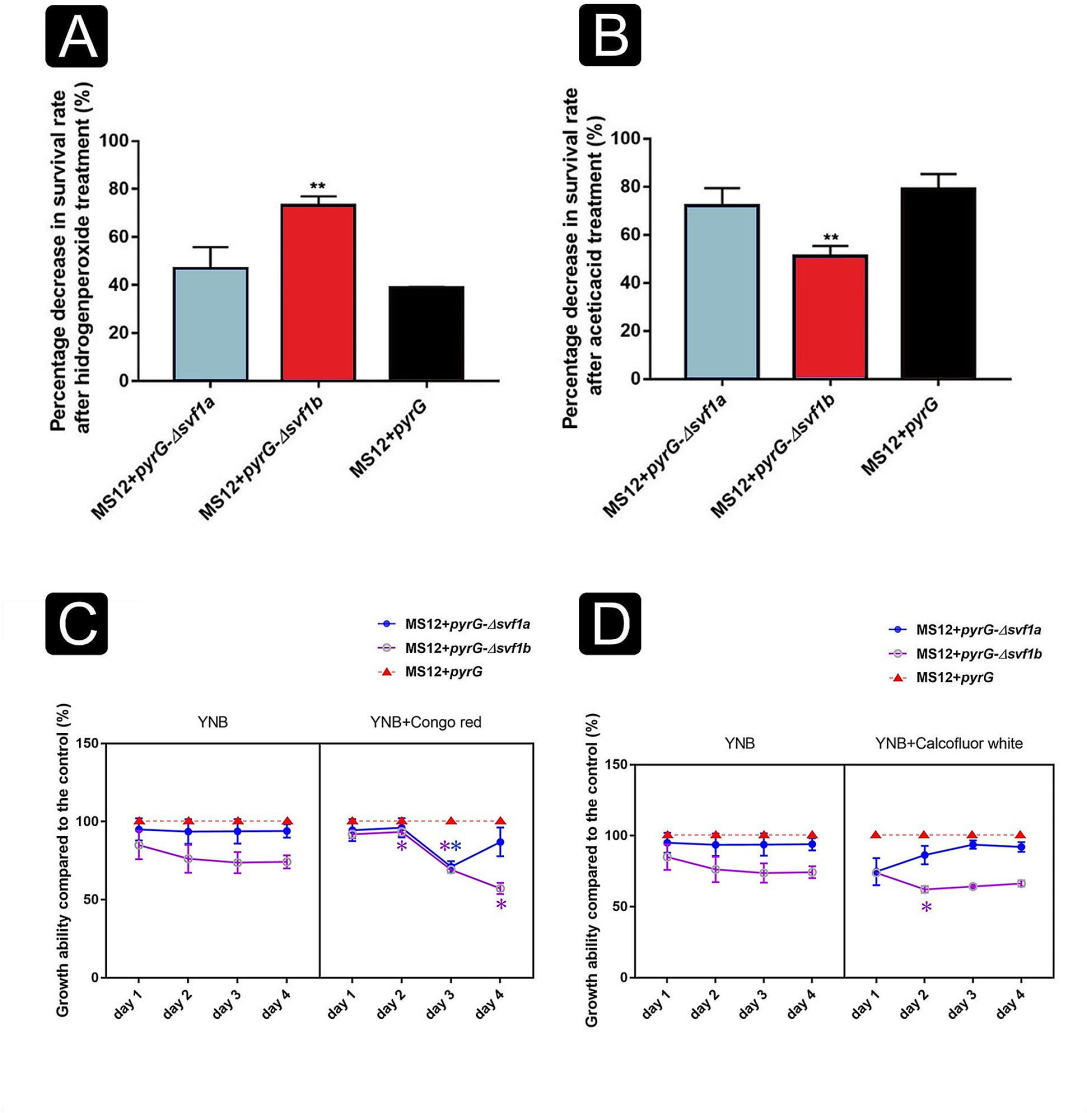
Spore survival of *svf1* mutant strains after treatment with acetic acid and hydrogen peroxide and effect of cell wall stressors to the growth of the *svf* mutants. The spore survival of *svf* mutant strains was evaluated following treatment with hydrogen peroxide (**A**) and acetic acid (**B**). The reduction in spore survival, expressed as a percentage, after exposure to these stressors was compared with the untreated condition. Growth of the *svf* mutants in the presence of CR (**C**) and CFW (**D**) stressors. Changes in growth of mutants were calculated by considering the growth of the *MS12 + pyrG* control as 100%. Values were calculated according to the two-sample unpaired *t* test statistical method. Values indicated by asterisks are significantly different from the value of the MS12 + *pyrG* strain measured at the same conditions (*P  *≤ *0.05; **P  *≤ *0.01).

Spores of the *Δsvf1b* mutant strain demonstrated increased susceptibility to hydrogen peroxide compared with the control strain, MS12 + *pyrG* (Fig. 5A). Conversely, spores of the same mutant exhibited an increased resilience to acetic acid treatment, in contrast to the control strain (Fig. 5B). The growth of the *Δsvf1a* mutant (MS12 *+ pyrG*-Δ*svf1a*) displayed a pronounced reduction on the third day of cultivation when exposed to the CR cell wall stressor. Notably, the *Δsvf1b* mutant exhibited a remarkable susceptibility to CR, resulting in a pronounced and indicating the value of significance in growth (Fig. 5C). At the same time, CFW exerted no statistically significant impact on the two mutants (Fig. 5D).

### Sphingolipid composition of the *svf1* mutants

**Fig 6:**
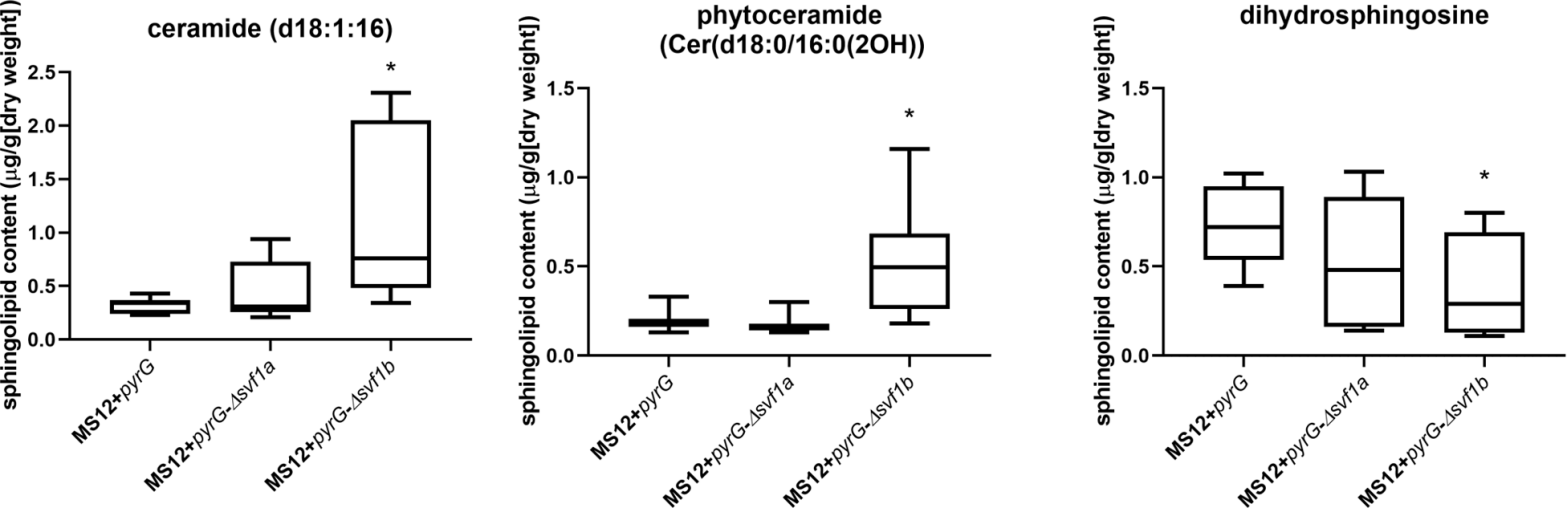
Sphingolipid composition of MS12 + *pyrG*, MS12 + *pyrG*-Δ*svf1a*, and MS12 + *pyrG*-Δ*svf1b* strains. The presented values are averages; amounts of sphingolipids were measured from at least six independent cultivations (error bars indicate standard deviations). Values followed by asterisks significantly differed from the corresponding value of the MS12 + *pyrG* strain according to the unpaired *t* test (*P  ≤  0.05).

Analysis of the sphingolipid profile revealed a strikingly altered sphingolipid composition in the *svf1b* knockout mutant compared with the control strain and the *Δsvf1a* mutant (Fig. S3). The absence of the *svf1a* gene did not affect the sphingolipid biosynthesis. In the MS12 + *pyrG*-Δ*svf1b* isolates, ceramide and phytoceramide content significantly increased while that of the dihydroceramide significantly decreased (Fig. 6).

### Knockout of the *M. lusitanicus svf1* genes affects the transcription of genes participating in lipid homeostasis and synthesis

In the *M. lusitanicus* genome, two hypothetical Ypk proteins were found by a similarity search using the amino acid sequence of the *S. cerevisiae* Ypk1 (UniProt ID: P12688), which is a protein kinase required for plasma membrane lipid and protein homeostasis (34). The identified proteins were named Ypka (Protein ID: 1490516) and Ypkb (Protein ID: 1472422). Relative transcript level of *ypka* was significantly decreased in the MS12 + *pyrG*-Δ*svf1b* mutant while relative transcript level of *ypkb* did not alter in the mutants (Fig. S4A).

Based on similarity searches using the *S. cerevisiae* Lcb3 (long-chain base phosphatase; UniProt ID: P47013) and Lcb4 (sphingoid long-chain base kinase; UniProt ID: A0A6C1E1U5) amino acid sequences, one putative *lcb3* (protein ID: 1517363) and three putative *lcb4* genes were identified in the *M. lusitanicus* genome, respectively. The three *lcb4* genes were named *lcb4a* (protein ID: 1515220), *lcb4b* (protein ID: 1322836), and *lcb4c* (protein ID: 1472618). Relative transcript level of *Mucor lcb3* was found to be significantly increased in the MS12 + pyrG-Δ*svf1b* strain (Fig. S4B). Knockout of both *svf1a* and *svf1b* was associated with a significantly decreased transcription of *lcb4a* and a significantly increased transcription of *lcb4b*. The absence of the *svf1* genes had no effect on the relative transcript level of the *lcb4c* gene (Fig. S4C).

### *In vitro* interaction with J774.2 macrophages

To examine whether recognition and internalization of the fungal spores by macrophages were affected by the knockout of the *svf1* genes in *M. lusitanicus*, the phagocytosing capacity of J774.2 cells exposed to the control (MS12 + *pyrG*), the MS12 *+ pyrG*-Δ*svf1a,* and the MS12 *+ pyrG*-Δ*svf1b* strains was tested. There was no difference in the phagocytosis and the killing rate for the mutant and the control strains (Fig. S5 and S6).

### *In vivo* study of virulence of *svf* mutant strains

**Fig 7:**
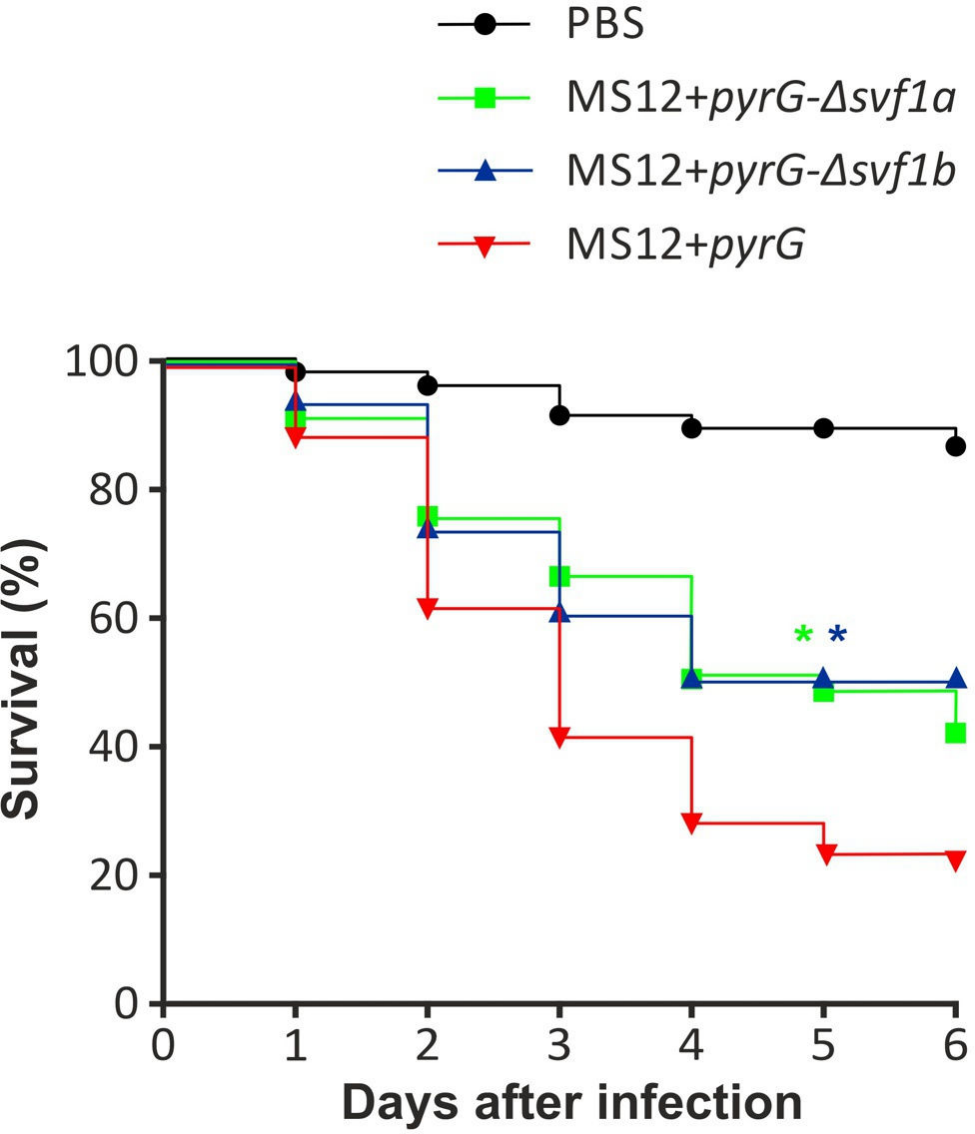
Investigation of the pathogenicity of *svf1* mutants. Oregon (wild type) *D. melanogaster* (*n* = 60) infected with *svf1* mutants and control *M. lusitanicus* MS12 *+ pyrG* strains. The results are summarized from three independent experiments. Survival curves marked with an asterisk were significantly different from the control strain according to the log-rank (Mantel-Cox) test. Values indicated by asterisks are significantly different from the value of the MS12 + *pyrG* strain measured at the same conditions (*P  *≤ *0.05).

To investigate the role of Svf1 proteins in the pathogenicity of *M. lusitanicus*, *Drosophila melanogaster* was used as a non-vertebrate animal model. In *D. melanogaster*, lack of the *svf1* genes significantly decreased the virulence in the mutants compared with the control (Fig. 7).

## DISCUSSION

Survival factor 1 proteins have been identified in some fungi, including *Aspergillus*, *Fusarium*, *Saccharomyces*, *Schizosaccharomyces,* and *Sclerotinia* species (1, 2, 4–7), where it has been found to be involved in the regulation of the responses to various (e.g., osmotic, oxidative, cold, or fungicide) stresses and may affect the virulence of pathogenic fungi (1–3, 6, 7). The genome of *M. lusitanicus* contains two putative Svf1-encoding genes, i.e., *svf1a* and *svf1b*. Both were found to be expressed, although the *svf1b* displayed higher transcription levels than *svf1a*. Expansion of genes is a known feature in Mucorales and thus in *M. lusitanicus* too (35).

Knockout of the gene resulted in significant changes in the growth and physiological properties of the fungus only in the case of *svf1b*. In *Mucor*, the effect of one of the multiple genes on the molecular processes of the cell has generally been found to be stronger than that of the others (36–39). In the case of the *svf1* genes, based on our experiments, expression of the *svf1b* gene seems to be determining regarding the survival factor function. Knockout of *svf1b* caused reduced colony growth and viability of spores. Effect of the Svf1 on spore production and viability was also observed in *A. nidulans* and *F. graminearum* (3, 5).

Temperature is known to be a critical environmental factor that influences the growth and development of filamentous fungi. Transcription of the *svf1* genes showed a temperature dependence as they were significantly upregulated at 20°C and downregulated at ≥30°C compared with the transcription at 25°C. Temporal cold stress caused the upregulation of both *svf1* genes while a 30-min heat stress increased the transcription level of only the *svf1b*. This pattern reinforces the differential and temperature-responsive expression of the two genes. In other fungi, Svf1 expression has also been associated with low temperature conditions (2, 3, 6). Moreover, deletion of the *svf1* genes caused increased sensitivity to cold stress in *S. cerevisiae*, *F. graminearum,* and *A. nidulans* (3, 5, 6). In case of *M. lusitanicus*, *svf1b* knockout also caused cold hypersensitivity and decreased colony size at lower temperatures, especially at <20°C, indicating the role of Svf1b in temperature-dependent growth regulation in *Mucor*.

Agents generating oxidative, cell wall, and membrane stress exerted various effects on *svf1* gene expression revealing a dynamic regulation of these genes. Moreover, acetic acid and hydrogen peroxide treatment caused a significant increase in the relative transcription level of both genes. Previously, *svf1* transcription also displayed high levels in the hyphae of *S. sclerotiorum* under oxidative stress (4). Knockout of *svf1b* caused increased sensitivity of *M. lusitanicus* to the cell wall stressor CR and the oxidative stress caused by hydrogen peroxide.

Alejandre-Castañeda et al. (40) recently described two transcription factors, Tec1 and Tec2, which participate in the regulation of oxidative metabolism and affect the hyphal growth and the virulence of *M. lusitanicus*. However, analysis of the promoter region of none of the *svf1* genes revealed the canonical Tec1-binding site (i.e., the 5ʹ-CATTCY −3′ sequence)

As carotenoids are potent antioxidants and may have a protective effect against oxidative stressors (41), the increased carotenoid content in the *Δsvf1b* mutant colonies can be consistent with the disturbed cellular response to oxidative stress in the lack of the Svf1b protein. Interestingly, *svf1b* knockout decreased the sensitivity of the fungus to acetic acid. The differential effect of Svf1b deficiency on the various stress response mechanisms indicates that Svf1b exerts its effects through the regulation of these processes, as it is also found for other fungi (11).

In *S. cerevisiae*, the TOR complex 2-dependent protein kinase Ypk1 is a central regulator of the lipid and sphingolipid content and composition in response to various membrane stresses (e.g., ROS stress, heat and cold shock, hypotonicity, and other stressful conditions) and participates in the maintenance of the growth and viability under these conditions (34, 42, 43). In *S. cerevisiae*, Svf1 is also required for optimal growth and survival. Moreover, the lack of a functional Svf1 resulted in phenotypic similarities to the *ypk1* knockout mutants suggesting that the Svf1-controlled pathways converge on a signaling cascade regulated by Ypk1 (10). In *M. lusitanicus*, transcription of *ypka* significantly decreased in the *svf1b* knockout mutant raising a similar role for and a possible link between *svf1b* and *ypk1a* in this fungus.

In yeast, the role of the Svf1 protein in the regulation of sphingolipid biosynthesis was proven (6, 10). It was found that *Saccharomyces* Svf1 regulates the generation of a specific subset of phytosphingosine. Although phytosphingosine content seems to be unchanged, knockout of *svf1b* had a radical effect on the sphingolipid composition of *M lusitanicus*. Dihydroceramide and ceramide accumulation suggest that the formation of ceramide derivatives and later biosynthetic steps damaged in the mutant.

In eukaryotes, long-chain bases (i.e., dihydrosphingosines and phytosphingosines) are the precursors of ceramides (44, 45). In *S. cerevisiae*, the Lcb4 sphingoid long-chain base kinase and Lcb3 long-chain base phosphatase have a role in the phosphorylation and dephosphorylation of dihydrosphingosine and phytosphingosine and participate in the incorporation of exogenous long-chain bases into the ceramide biosynthesis (44, 46). The yeast Lcb3 was found to be regulated by Ypk1 and Svf1 (10, 40), and it was suggested that Svf1 acts in concert with Lcb3 and Lcb4 (10). In *M. lusitanicus*, knockout of *svf1b* resulted in an increased transcript level of *lcb3* and accordingly accumulation of ceramide derivates. These results suggest a clear connection between *svf1b* and *lcb3* in *M. lusitanicus*. At the same time, the relative transcript level of *lcb4a* significantly decreased and that of the *lcb4b* significantly increased in *Δsvf1a* and *Δsvf1b* mutants. This result suggests that *svf1a* and *svf1b* had a connection with the two *lcb4* genes of *Mucor*. However, the oppositely changing expression of the two genes indicates that the regulation of the genes tries to compensate for the altered functioning caused by the loss of the *svf1* genes. The altered expression of the *lcb3* and *lcb4* genes may also be related to the cold sensitivity of the *svf1b* knockout mutant. In yeast, knockout of *lcb3* and *lcb4* resulted in a temperature-sensitive phenotype (47).

Knockout of both *Mucor* genes caused a decreased virulence in *D. melanogaster,* indicating that expression of both genes affects the pathogenicity of *Mucor. D. melanogaster* has been shown in many studies to be a suitable invertebrate model for testing the virulence of mucormycosis-causing fungi (48, 49). Previously, deletion of *Aspergillus svfA* also caused decreased pathogenicity in zebrafish and mouse models (5). It also should be mentioned that the decrease in virulence of the *svf1b* knockout mutant may be caused by a decrease in viability and stress tolerance rather than the lack of an independent virulence factor.

### Conclusion

Our studies indicate that the functions reported for fungal survival factor 1 proteins, such as their role in the maintenance of the viability, the oxidative and cold stress response, sphingolipid biosynthesis, or pathogenicity, can be primarily associated with Svf1b among the two Svf1 proteins of *M. lusitanicus*. Although the expression of *svf1a* was also influenced by certain stresses (i.e., certain temperatures, cold and heat stress, cell wall and membrane stressors, hydrogen peroxide, acetic acid, and metal ions), and the absence of the gene reduced the sporulation capacity and the virulence, further studies are required to clarify the role of this gene, which seems to be only partially overlapping with that of the *svf1b* as knockout of *svf1a* had no significant effect on the sphingolipid biosynthesis and viability. Based on experiments in *A. nidulans*, Lim et al. (5, 11) proposed Svf1 as a novel master regulator of growth, morphogenesis, and secondary metabolism, which affects the expression of other regulatory proteins. The fact that the lack of Svf1b had such a wide-ranging effect on the growth, spore formation, viability, carotenoid and sphingolipid production, and virulence suggests a similar role for the protein in *Mucor* as well.

## MATERIALS AND METHODS

### Strains, media, and growth conditions

The *M. lusitanicus* MS12 strain, which is a leucine and uracil auxotrophic (*leuA*^−^ and *pyrG*^−^) derivative of the wild-type CBS277.49 (50) was used to construct the knockout strains. As the *pyrG* genotype slightly affects the growth of *M. lusitanicus,* the strain MS12 + *pyrG* (51), in which the uracil auxotrophy was complemented by the functional *pyrG* gene, was used as a control during the analysis of the mutants.

For nucleic acid extractions, 10^6^ sporangiospores were plated on solid minimal medium [YNB; 10 g/L glucose, 0.5 g/L base of yeast nitrogen without amino acids (BD Difco, Becton Dickinson, Franklin Lakes, NJ, USA), 1.5 g/L (NH_4_)_2_SO_4_, 1.5 g/L sodium glutamate, and 20 g/L agar] supplemented with leucine and/or uracil (0.5 mg/mL), if necessary, and incubated for 2 or 4 days at 25°C. For RNA extraction after cultivating the fungus in the presence of a stressor, solid YNB medium was supplemented with 2 mg/mL CR, 0.1 mg/mL CFW, 0.004% (m/vol) SDS, 0.1% (vol/vol) TRX, 0.5 mM CuSO_4_, 1.5 mM FeSO_4_, or 0.5 mM ZnSO_4_. In case of acetic acid and hydrogen peroxide, the stressors (60 and 10 mM, respectively) were added to liquid YNB broth.

For RNA extraction after cold and heat stress, 10^6^ sporangiospores were inoculated in liquid minimal medium (i.e., YNB broth without agar) and cultivated for 48 hours at 25°C. Then, to induce cold and heat stress, the cultures were transferred for 30 min at 4 or 50°C. After incubation, the mycelia were collected for subsequent RNA extraction.

To test the mitotic stability of the transformants, malt extract agar (MEA; 10 g/L glucose, 5 g/L yeast extract, 10 g/L malt extract, and 20 g/L agar) was used as a complete, non-selective medium.

To examine the effect of the temperature on the growth, 10^4^ spores were point-inoculated at the center of solid YNB plates and incubated at 14, 18, 25, and 35°C. To determine the effect of different stressors, the same amounts of spores were plated onto solid YNB supplemented with CR, CFW, SDS, TRX, CuSO_4_, FeSO_4_, or ZnSO_4_ in the same concentrations as described above.

### Susceptibility tests

Sensitivity of the fungal strains to different heavy metals was examined in a 96-well microtiter plate assay. The susceptibility test was performed in three biological replicates. The final concentration of FeSO_4_ and CuSO_4_ in the wells ranged from 0.5 to 5 mM, whereas the final concentration of MgSO_4_ in the wells ranged from 50 to 500 mM. Inocula were prepared and diluted in liquid YNB. Plates were incubated for 48 hours at 25°C.

### Molecular techniques

Genomic DNA and total RNA were isolated using ZR Fungal/Bacterial DNA MiniPrep (Zymo Research, Irvine, CA, USA) and Direct-zol RNA MiniPrep (Zymo Research, Irvine, CA, USA) kits, respectively, according to the manufacturer’s instructions. To amplify genes or gene fragments from genomic DNA, the Phusion High Fidelity DNA Polymerase (Thermo Scientific, Waltham, MA, USA) was used according to the manufacturer’s recommendations. The PCR products were isolated and concentrated using the Zymoclean Large Fragment DNA Recovery Kit (Zymo Research, Irvine, CA, USA) and DNA Clean & Concentrator-5 (Zymo Research, Irvine, CA, USA). The primers used in the study are listed in Table S2.

### Quantitative real-time reverse transcription PCR analysis

Reverse transcription was carried out with the Maxima H Minus First Strand cDNA Synthesis Kit (Thermo Scientific) using random hexamer and oligo (dT)18 primers, following the manufacturer’s instructions. The qRT-PCR experiments were performed in a CFX96 real-time PCR detection system (Bio-Rad) using the Maxima SYBR Green qPCR Master Mix (Thermo Scientific) and the primers presented in Table S2. Relative quantification of copy number and gene expression was performed using the threshold cycle (2^−ΔΔCT^) method (52) using the *M. lusitanicus* actin gene as a reference (53). The amplification conditions involved 95°C for 3  min followed by 40 cycles at 95°C for 15  s, 60°C for 30  s, and 72°C for 30  s. All experiments were performed in biological and technical triplicates.

### Knockout of the *svf1* genes of *M. lusitanicus*

Gene knockout was carried out by exchanging the coding regions of the *svf1* genes with a functional *pyrG* gene (CBS277.49. v2.0 genome database ID Mucci1.e_gw1.3.865.1). This was achieved by CRISPR-Cas9-mediated homology-directed repair (HDR) following the method and strategy described previously (39, 54).

The protospacer sequences designed to target the DNA cleavage in the *svf1a* and the *svf1b* genes were the following, 5′- TGAAAACAATGTTGAGATGT - 3′ and 5′ - CGACTTCTTCAACATTCAGC - 3′, respectively. Alt-R CRISPR RNA (crRNA) and Alt-R CRISPR-Cas9 transactivating crRNA molecules (tracrRNA) were designed and purchased from Integrated DNA Technologies (IDT, Coralville, IA, USA). To form the crRNA:tracrRNA duplexes (i.e., the guide RNAs [gRNAs]), IDT nuclease-free duplex buffer (IDT, Coralville, Iowa, USA) was used according to the manufacturer’s instructions.

Disruption cassettes functioning also as the template DNA for the HDR were constructed by PCR using the Phusion Flash High-Fidelity PCRMaster Mix (Thermo Scientific). At first, two fragments, upstream from start codon and downstream from stop codons of the targeted gene and the *M. lusitanicus pyrG* gene along with its own promoter and terminator sequences, were amplified using gene-specific primer pairs (Table S2). The amplified fragments were fused in a subsequent PCR using nested primers (Table S2); the ratio of the fragments in the reaction was 1:1:1. Fig. S7 shows the genome-editing strategy used in this study.

Template DNA (5 µg), gRNA (10 µM), and the Cas9 nuclease (10 µM; Alt-R S.p. Cas9 Nuclease, IDT, Coralville, IA, USA) were introduced together into the *M. lusitanicus* MS12 strain by PEG-mediated protoplast transformation as described previously (39, 54). Transformants were selected on solid YNB medium by the complementation of the uracil auxotrophy of the MS12 strain; from each primary transformant, monosporangial colonies were formed under selective conditions. Knockout of the *svf1* genes and the presence of the integrated *pyrG* gene were proven by PCR using the primers listed in Table S2 (Fig. S8). Mutants, which were named MS12 *+ pyrG*-Δ*svf1a* and MS12 *+ pyrG*-Δ*svf1b*, proved to be mitotically stable retaining the integrated fragment even after 20 cultivation cycles. qRT-PCR analysis proved the absence of the transcripts of the disrupted genes. For further analysis two independently derived mutants were selected. qRT-PCR analysis indicated the lack of the *svf1a* and *svf1b* transcripts in the transformants.

### Sporulation capacity test

Sporangiospores (10^4^) were point-inoculated onto MEA plates and incubated at 25°C for 4 days. Spores were then collected in 1× sterile phosphate-buffered saline [PBS; 137  mM NaCl, 2.7  mM KCl, 10  mM Na_2_HPO_4_, 2  mM KH_2_PO_4_ (pH 7.4)] and centrifuged for 10  min at 4°C at 2,000  ×  *g*. Spore concentration was determined by Bürker chamber counting.

### Viability assay

10^6^ spores were treated with 10 mM hydrogen peroxide or 60 mM acetic acid for 30 min at 4°C in liquid YNB. Subsequently, 100 spores were plated onto YNB medium and incubated at 25°C for 2 days. The number of colony forming units (CFU) was determined after 24 h of incubation. CFU of the untreated control strain was taken as the 100% viability.

### Measurement of the total carotenoid content

Extraction of carotenoids was performed according to Papp et al. (55). Total carotenoid content was measured by spectrophotometry at 450 nm, as described elsewhere (53).

### Analysis of the sphingolipid composition

For sphingolipid extraction, 10^6^ spores were inoculated in 10 mL liquid YNB medium and were shaken for 7 days at 25°C at 210 rpm on a heated shaker (IKA KS 4000). Then, the mycelium was separated from the medium using a vacuum filter, lyophilized, and stored at −20°C until extraction. Lipid extraction was performed in one step using ethyl acetate:2-propanol:water (60:30:10) according to Bielawski et al (56) with some modifications, as described. After the addition of 80 µL water to 20 mg freeze-dried mycelium, lipids were extracted with 1 mL of the triphasic solvent system by sonication for 10 min followed by centrifugation at 20,000 × *g* for 10 min. The supernatant was removed, and the extraction was repeated once in a similar way. The combined supernatants were evaporated to dryness under nitrogen, and the residue was dissolved in 200  µL of the HPLC mobile phase “B.” Liquid chromatography - high resolution mass spectrometry (LC-HRMS) measurements were performed using a DionexUltimate 3000 UHPLC system (Thermo Scientific) coupled to a Q Exactive Hybrid Quadrupole-Orbitrap Mass Spectrometer (Thermo Scientific) operating with a heated electrospray interface (HESI). Metabolites were separated on a Discovery HS C-18 column (150 × 2.1 mm, 3 µm) (Supelco, Bellefonte, PA, USA) thermostated at 45°C. Mobile phase A consisted of 5 mM ammonium acetate containing 0.1% acetic acid, whereas methanol/2-propanol (1/1) containing 5 mM ammonium acetate and 0.1% acetic acid served as mobile phase B. Gradient elution program was applied as follows: 0–3 min: 75% B, 3–38 min: 100% B, 38–53 min: 100% B, 53–54 min: 75% B, 54–60 min: 75% B. The flow rate was kept at 0.2 mL/min, and the injection volume was 5 µL. All samples were analyzed in both positive and negative ionization modes using the following ion source settings: probe heater temperature and ion transfer capillary, spray voltage, sheath gas flow rate, auxiliary gas flow rate, and S-lens RF level were set at 300°C, 350°C, 3.75 kV, 35 arbitrarily, 10 arbitrarily, and 50 arbitrarily, respectively. For data acquisition, the full scan/data-dependent MS/MS method (full MS/ddMS2) was applied, where the full scan MS spectra were acquired at a resolution of 70,000 from *m/z* 100 to 1,200 with a maximum injection time of 100 ms. For each full scan, 10 ddMS2-scans were performed with a resolution of 17,500 and a minimum automatic gain control target of 1.00 × 10^5^. The isolation window was 1 *m/z*. Instrument control and data collection were carried out using Trace Finder 4.0 (Thermo Scientific) software. The raw data files were processed with Compound Discoverer 2.1 software for chromatographic alignment, compound detection, and accurate mass determination. Lipids were identified based on their exact mass and MS/MS fragmentation patterns. The experiment was carried out in three biological and 2–2 technical parallel experiments.

### Phagocytosis assay

The murine macrophage-like cell line J774.2 (Invitrogen) was cultured in DMEM (Lonza) supplemented with 10% heat-inactivated FBS (Fetal Bovine Serum, Biosera) and 1% 100 × penicillin-streptomycin solution (Lonza) at 37°C, 5% CO_2_ and 100% relative humidity. J774.2 macrophages (1 × 10^5^  cells/mL) were seated on a 24-well plate 16 h before the experiment. Four hours before infection, cells were stained with CellMask deep red plasma membrane stain (Thermo Scientific). Briefly, 0.5 µL CellMask Deep Red Plasma Membrane stain was added to each well of the plate and incubated in the dark for 10 min at 37°C, 5% CO_2_, and 100% relative humidity. The cells were then washed three times with PBS and incubated for 4 h in 1 mL/well fresh medium. Subsequently, fungal spores were collected from 1-week-old MEA cultures and stained with fluorescein-isothiocyanate (FITC, Sigma-Aldrich). Briefly, 2 × 10^7^ spores were pelleted, then resuspended in 0.1 mg/mL FITC solution, and incubated in the dark for 15 min at room temperature with continuous shaking. Then, the spores were washed three times with PBS, resuspended in 1 mL PBS, and the densities of the spore suspension were adjusted to the appropriate concentration. Four hours after macrophage staining, stained fungal spores were added to J774.2 cells at a multiplicity of infection (MOI) of 1:5 (cells:spores). Co-cultures were incubated for 3 h to allow phagocytosis. At the end of the incubation period, macrophages were gently scraped using a cell scraper and suspended in a single-cell suspension by pipetting. For analysis, collected samples were centrifuged with 200 g for 15 min, then resuspended in 200  µL PBS supplemented with 0.05% Tween-20 (Reanal) and measured on a FlowSight instrument (Amnis). Data were analyzed using the IDEAS software (version 6.2, Amnis). The number of engulfed cells was determined by examining 200 hundred pictures of individual macrophages, whereas phagocytic index (PI) was determined using the following formula:

PI = [mean spore count per phagocytic cells] × [% of phagocytosing cells containing at least one fungal spore].

### Survival assay in macrophages

J774.2 cells were seeded in 24-well plates at a density of 10^5^ cells/well in 1 mL DMEM and were infected with freshly collected fungal spores at a MOI of 5:1. Untreated control wells containing 1 mL DMEM, but no macrophages, were inoculated with the same number of spores. After 3, 4, 5, and 6 h of incubation, media were collected from each well, macrophages were lysed with sterile distilled water, and the suspensions were collected to the corresponding tubes again. Serial dilutions were prepared from each suspension. Finally, diluted spore suspensions were plated on MEA to quantify the CFU. Survival of spores was calculated after incubating the plates for 24 h at 25°C using the following formula:


$$Survival(\%)=CFU_{infected}\times 100/CFU_{control},$$

where CFU_infected_ is the CFU value of samples co-incubated with macrophages, whereas CFU_control_ is the CFU value of control samples, incubated in the same conditions, but without macrophages.

### Survival assay in *D. melanogaster*

The Oregon R *Drosophila* strain, originally obtained from the Bloomington Drosophila Stock Centre (Bloomington, IN, USA), was used throughout the experiments, which were performed as described previously (39). Spore suspensions were prepared in sterile PBS from 7-day-old cultures grown on YNB plates supplemented with 0.5 g/L of leucine at 25°C. Infection was carried out by dipping a thin needle in a suspension of fungal conidia (10^7^ conidia/mL) or PBS as the uninfected control, and subsequently, the thorax of the anesthetized fly was collected. The flies were counted at different times to monitor survival. The flies were moved into fresh vials every 2 days. Each experiment was carried out with 45 flies. The results shown are representative of three independent experiments.

### Statistical analysis

All measurements were made in at least two technical and three biological replicates. Statistical significance was analyzed using *t* tests or one-way analysis of variance (ANOVA) followed by Dunnett’s multiple comparison test using GraphPad Prism 7.00 (GraphPad Software, La Jolla, CA USA) as appropriate. *P* values less than 0.05 were considered statistically significant.

## SUPPLEMENTAL MATERIAL

Supplemental figures and tables spectrum.01103-24-s0001.pdf Fig. S1-S8; Tables S1 and S2.

ASM does not own the copyrights to Supplemental Material that may be linked to, or accessed through, an article. The authors have granted ASM a non-exclusive, world-wide license to publish the Supplemental Material files. Please contact the corresponding author directly for reuse.
