# Supplementary material for: Functional characterization of two survival factor 1 genes in Mucor lusitanicus
Source: Microbiol Spectr. 2024 Aug 27;12(10):e01103-24. doi: 10.1128/spectrum.01103-24 (PMC11448193; doi:10.1128/spectrum.01103-24)
Supplement: Supplemental figures and tables — Fig. S1-S8; Tables S1 and S2. [file spectrum.01103-24-s0001.pdf]

Supplemental files

Survival factor 1b participates in the regulation of sphingolipid content and stress response of *Mucor lusitanicus*

Olivér Jáger[a,b*,¶], Csilla Szebenyi,[a,b,¶] Tammam Abu Saleem[b], Anna Molnár[a,b], Vanda Kovács[a,b], Karina Kiss[a,b], Mónika Homa[a,b], Bernadett Vágó[a,b], Sándor Kiss-Vetráb[a,b], Mónika Varga[a], Rita Sinka[c], Csaba Vágvölgyi[a,b], Gábor Nagy[a,b,d#¶], Tamás Papp[a,b,d#¶]

#Address correspondence to Tamás Papp, pappt@bio.u-szeged.hu or Gábor Nagy nagy.gabor.04@szte.hu.

¶These authors contributed equally to this work. Author order was determined both alphabetically and in order of increasing seniority.

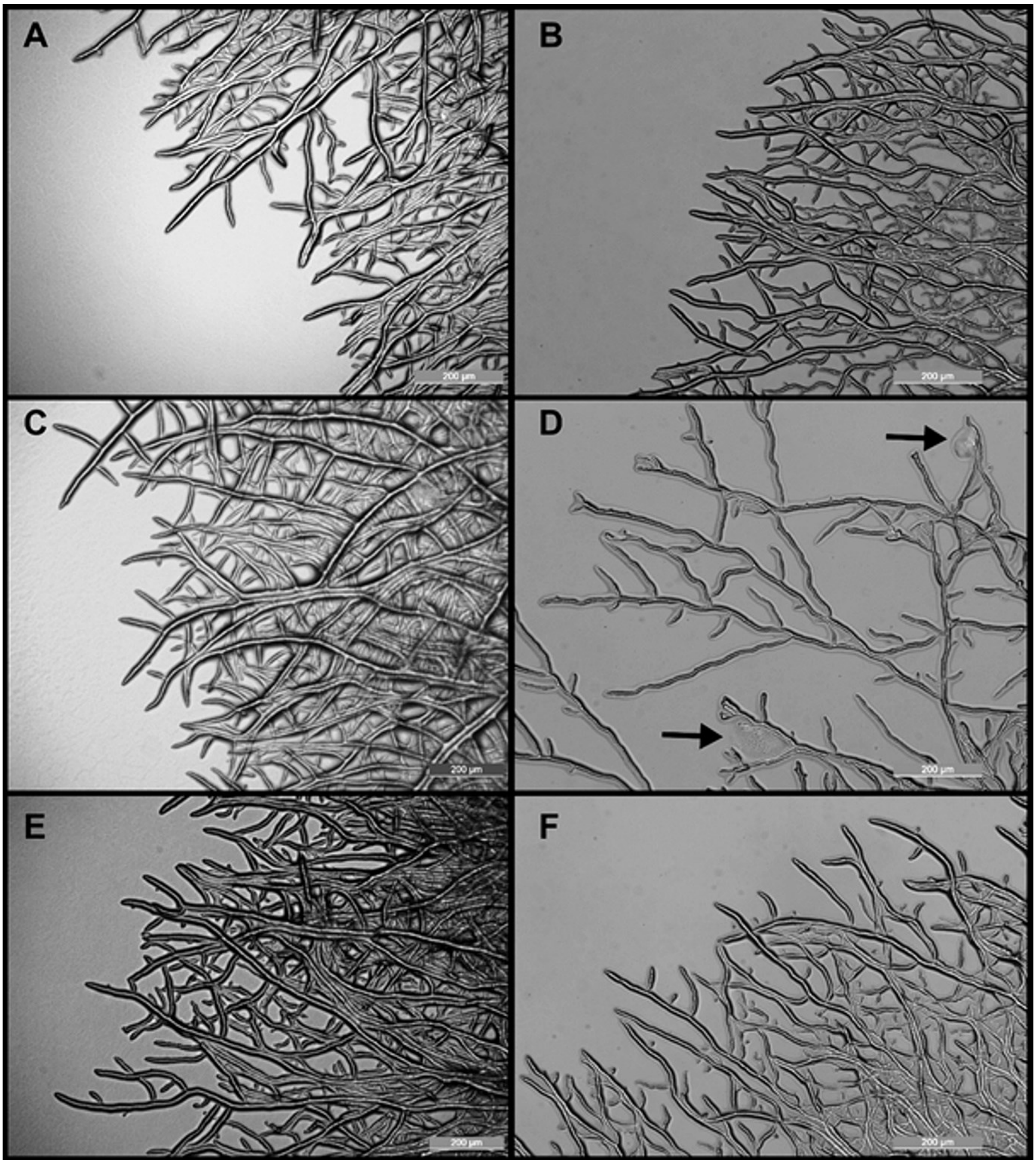

FIG S1 Micromorphology of *Mucor lusitanicus* strains after incubation at different temperatures. MS12+*pyrG*-Δ*svf1a* at 25 (**A**) and 14 °C (**B**); MS12+*pyrG*-Δ*svf1b* at 25 (**C**) and 14 °C (**D**); MS12+*pyrG* (control) at 25 (**E**) and 14 °C (**F**). Cytoplasmic releases in MS12+*pyrG*-Δ*svf1b* cells are indicated by arrows. Scale bar indicates 200 µm.

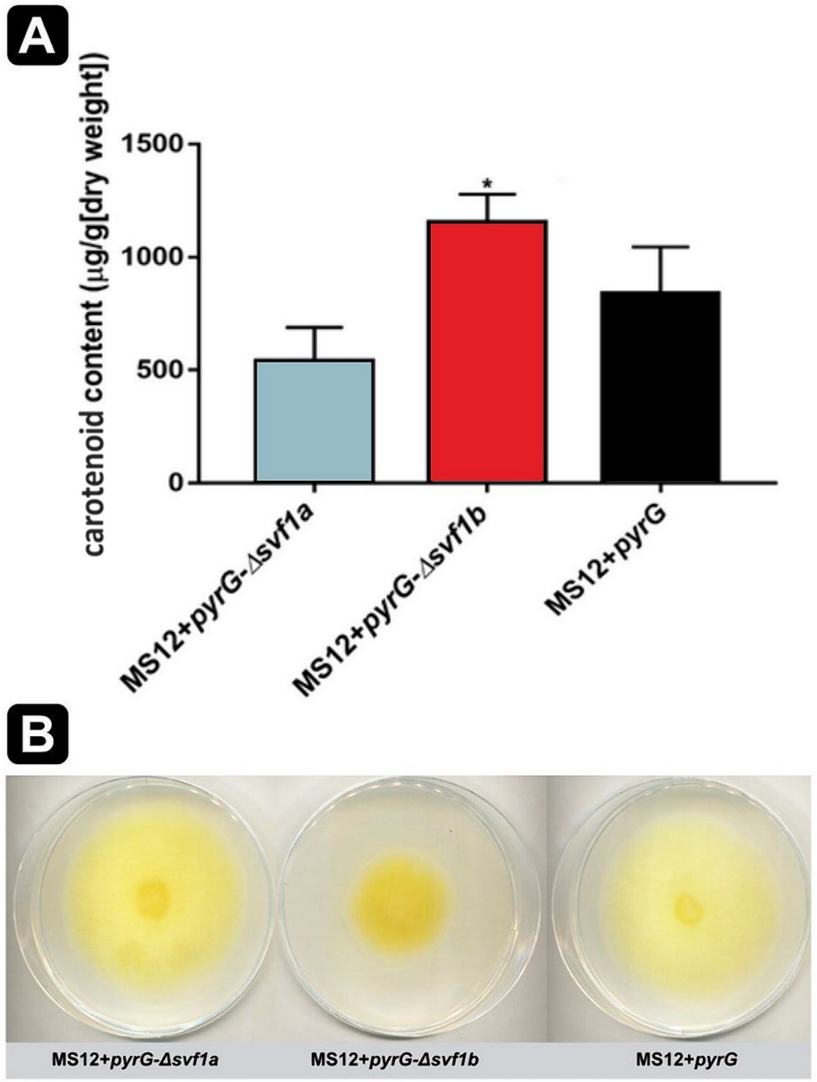

FIG S2 Effect of the *svf1* gene knockout on the carotenoid content of *M. lusitanicus*. Total carotenoid content in the mycelium of *svf1* mutants on the 4th day of cultivation (**A**). Colony color of the *svf1* mutants and the control strain on on the 4th day of cultivation on YNB medium (**B**). The presented values are averages of three independent experiments; error bars indicate standard deviation. Values followed by * significantly differed from the value of the control according to the unpaired t-test ($P \leq 0.05$).

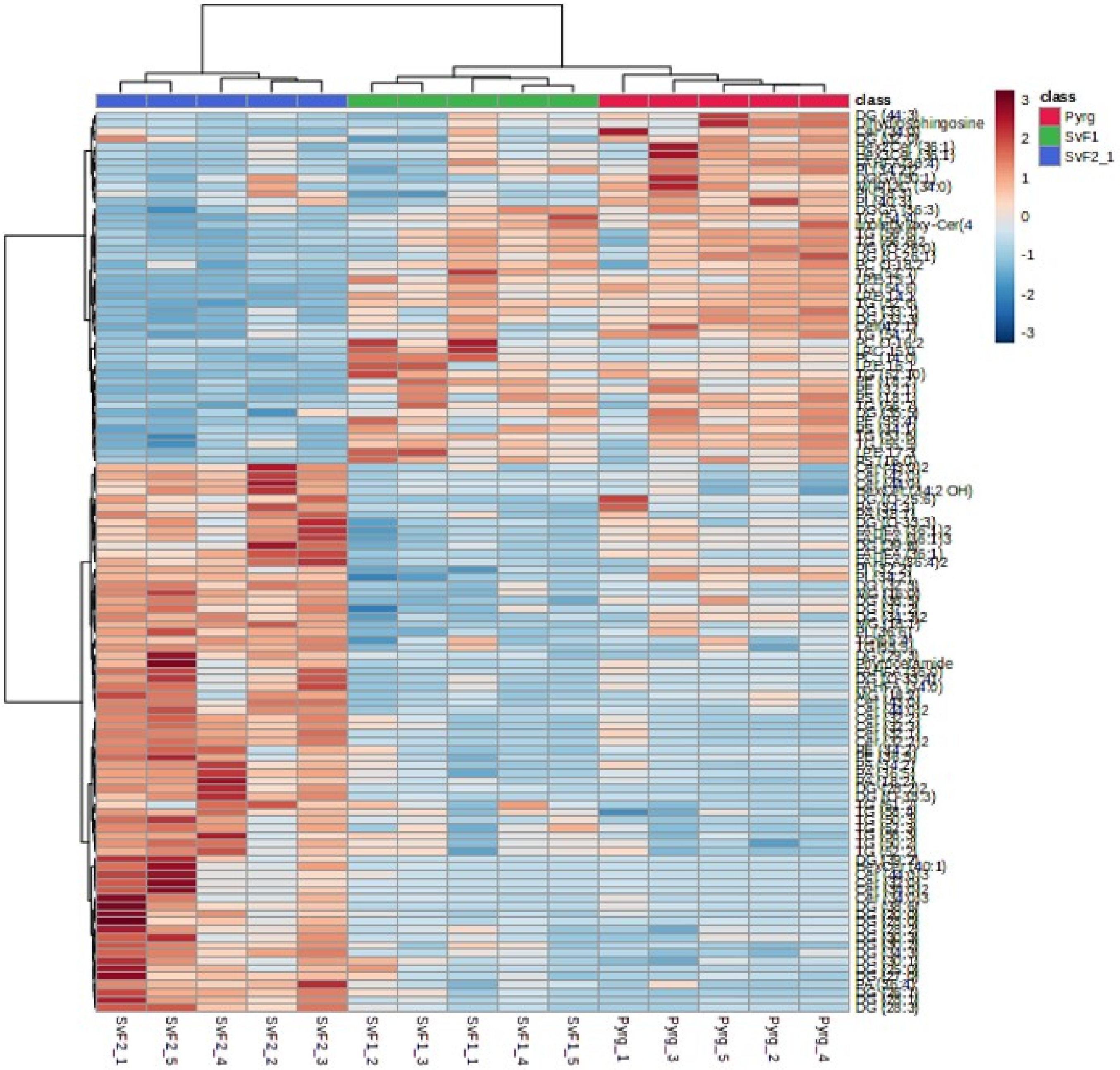

FIG S3 Heatmap representing the lipid composition of the *svf1* knock-out mutants and the control MS12+*pyrG* strain of *M. lusitanicus*. Data of five cultivations and extractions for each strain are represented as % of peak area to lipid mass; blue and red color depict the highest and lowest values, respectively. pyrG – MS12+*pyrG*, SvF1 – MS12+*pyrG*-Δ*svf1a*, SvF2 – MS12+*pyrG*-Δ*svf1b*.

FIG S4

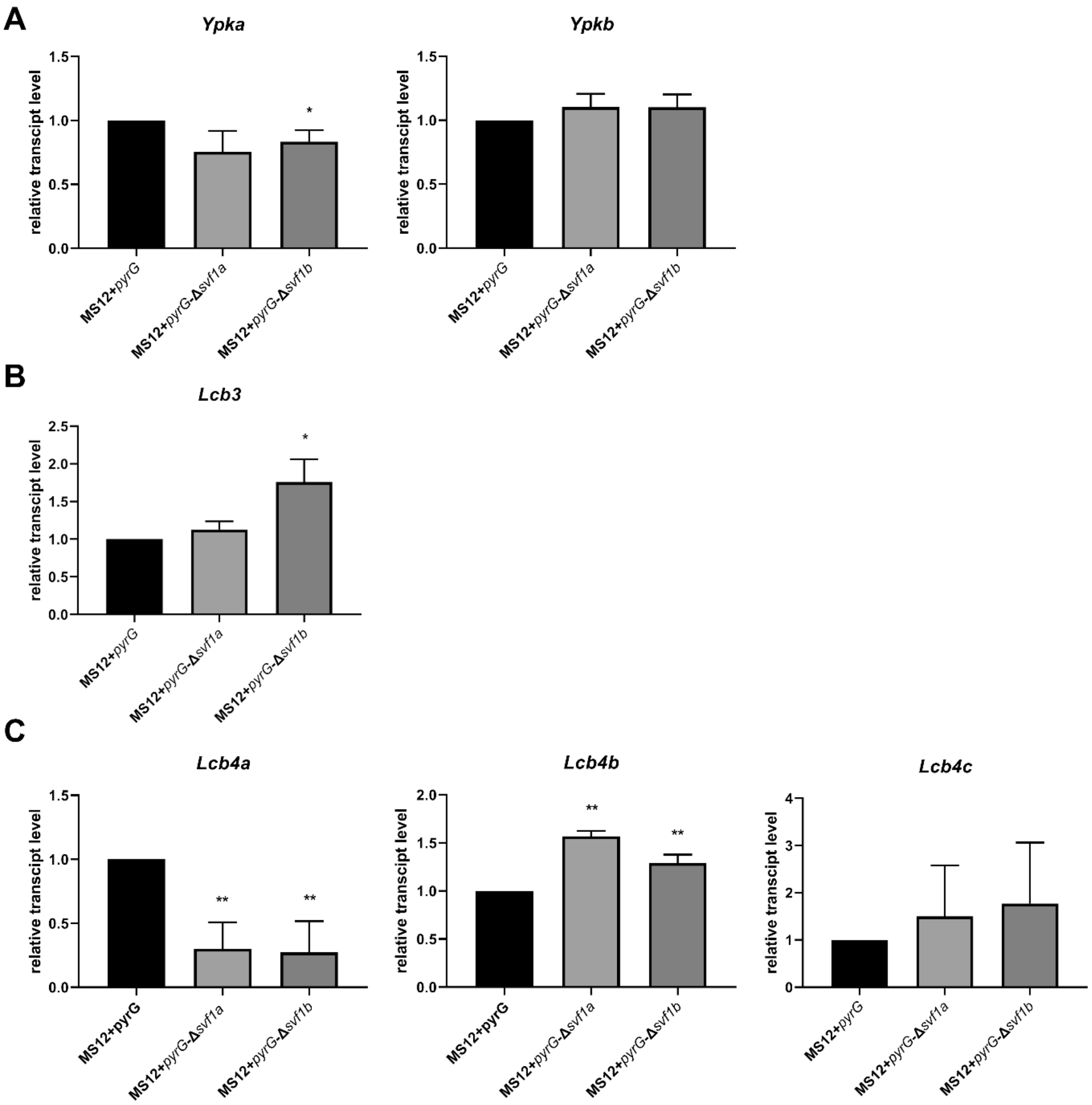

FIG S4 Relative transcript levels of the genes participating in the lipid homeostasis and synthesis, *ypk* **(A)**, *lcb3* **(B)**, and *lcb4* **(C)**. Strains were grown on YNB medium at 25°C; the transcript level of each gene measured in the MS12+*pyrG* control strain was taken as 1. The presented values are averages of three independent experiments of two independent isolates per mutant; error bars indicate standard deviations. Relative transcript values followed by asterisks were significantly differed from the untreated control according to the unpaired t test (*, P < 0.05; **, P < 0.01).

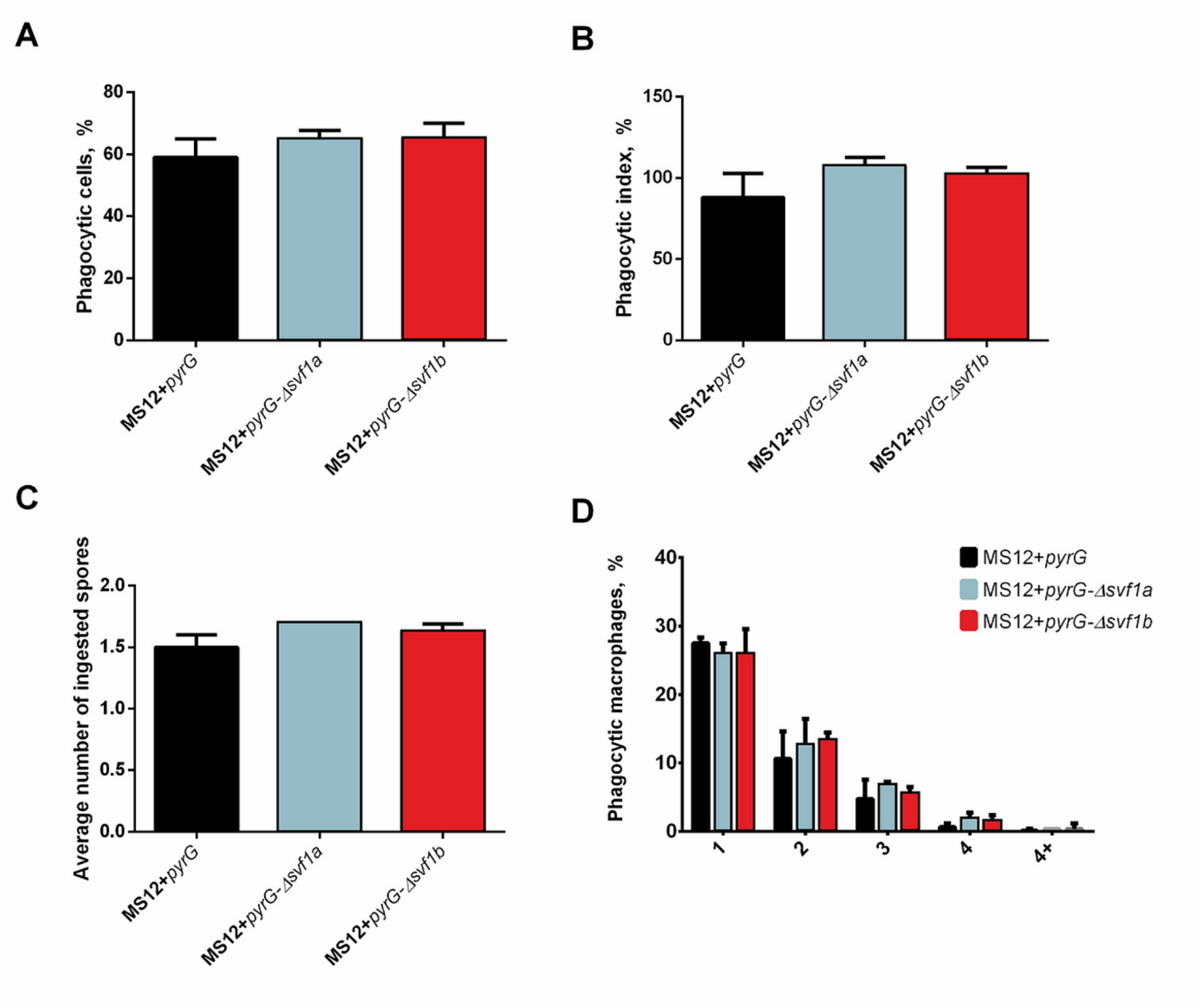

FIG S5 Results of the phagocytosis assay. (A) Percentage of phagocytic J774.2 cells after three hours of co-incubation with the MS12+*pyrgG*, MS12+*pyrG-Δsvf1a* and MS12+*pyrG-Δsvf1b* strains. **(B)** The mean phagocytic index values of macrophages challenged with the spores of the MS12+*pyrgG* and the mutant fungal strains. **(C)** The average number of MS12+*pyrgG*, MS12+*pyrG-Δsvf1a* and MS12+*pyrG-Δsvf1b* spores engulfed by phagocytic macrophages at three hours post-infection. **(D)** The phagocytic capacity of macrophages, that is the percentage of macrophages internalizing 1, 2, 3, 4 or more than 4 fungal spores per cell. Data are representative of three experiments.

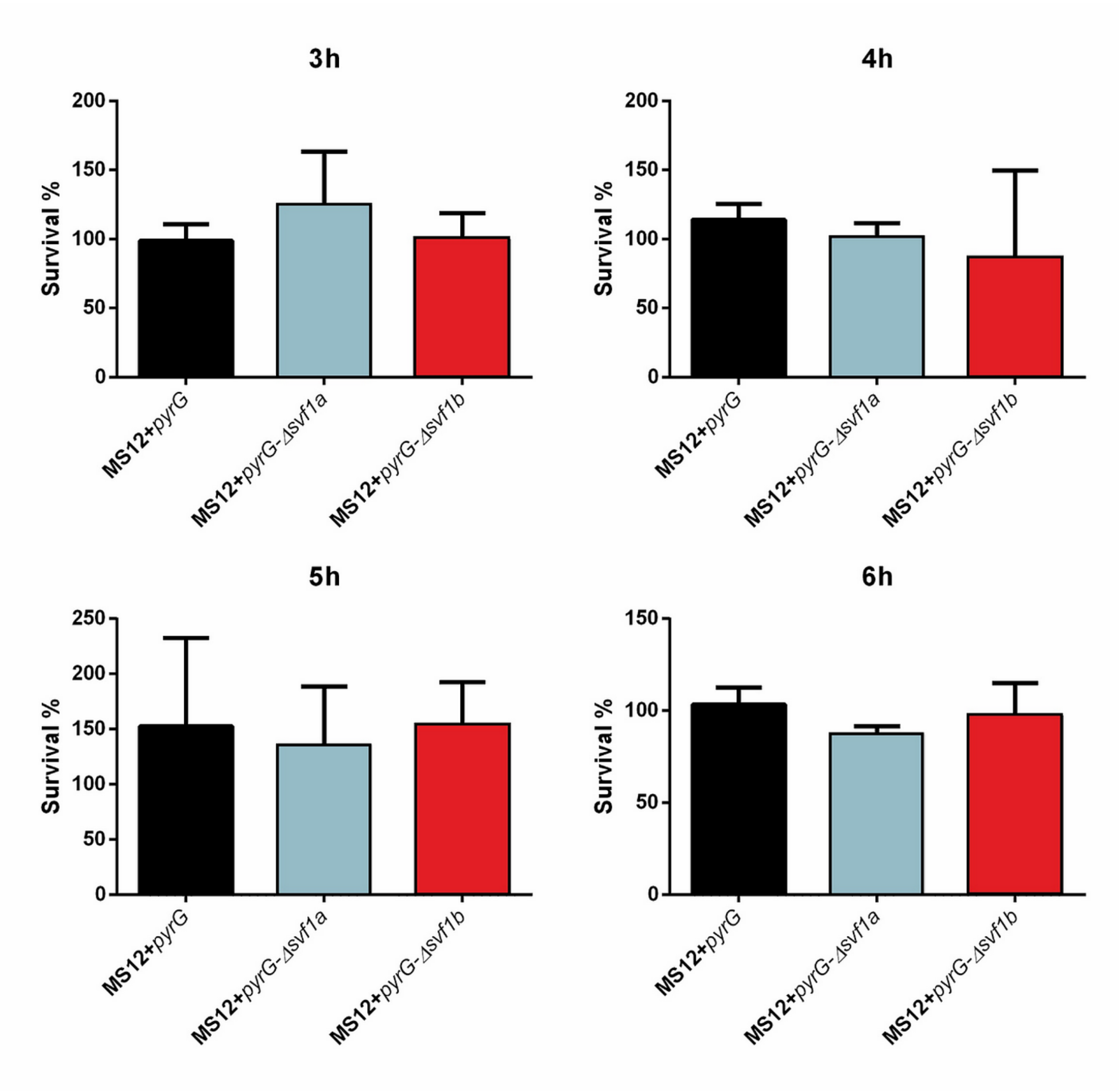

FIG S6 Intracellular survival of MS12+*pyrgG*, MS12+*pyrG-Δsvf1a* and MS12+*pyrG-Δsvf1b* spores in J774.2 murine macrophages. Survival of spores were determined at four different time-points, i.e. three, four, five and six hours post-infection. The presented results are representative of three independent experiments.

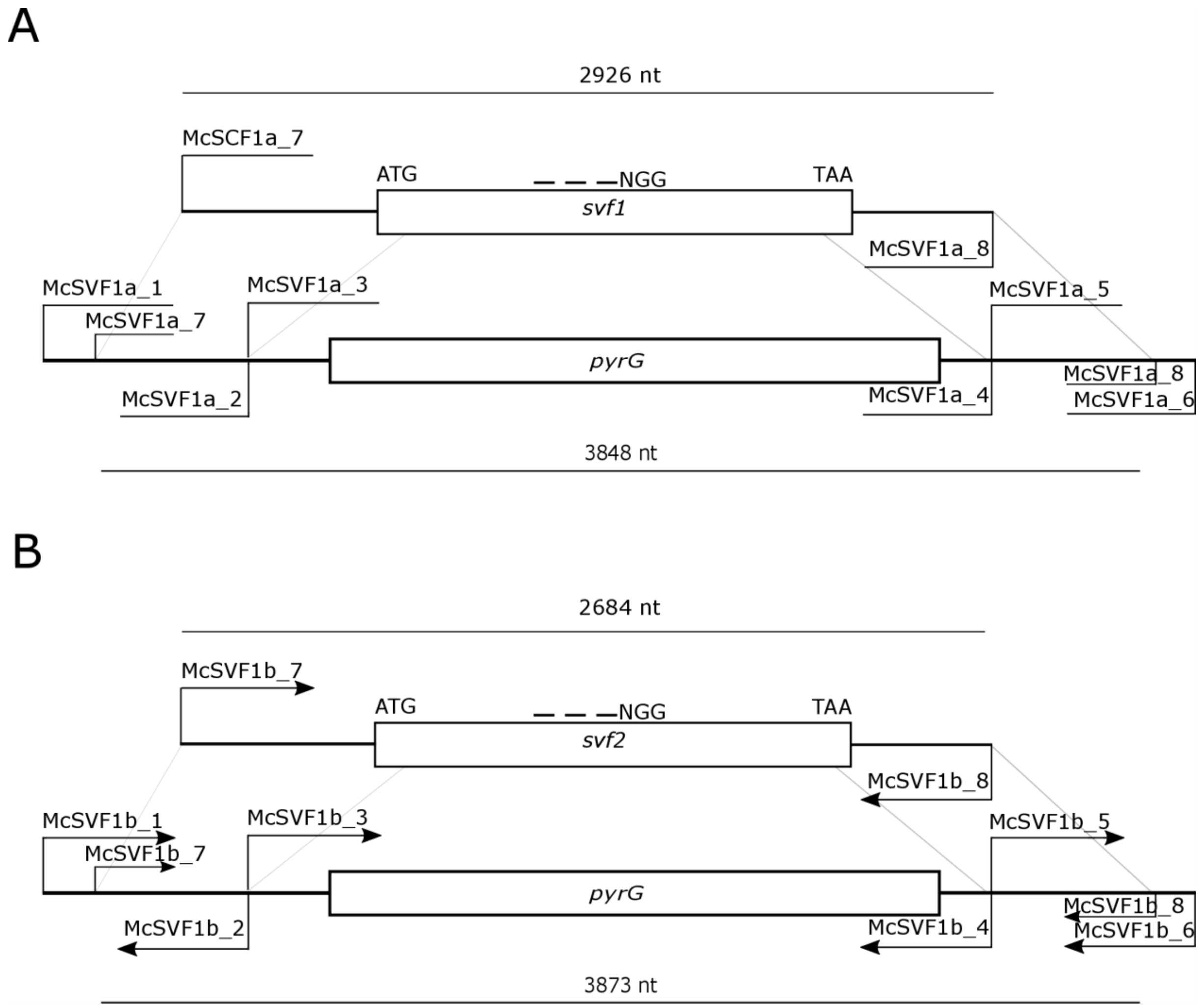

FIG S7 Genome editing strategy designed to disrupt the *svf1a* **(A)** and *svf1b* **(B)** genes of *Mucor lusitanicus* by the CRISPRCas9 method and positions of the primers used in the study.

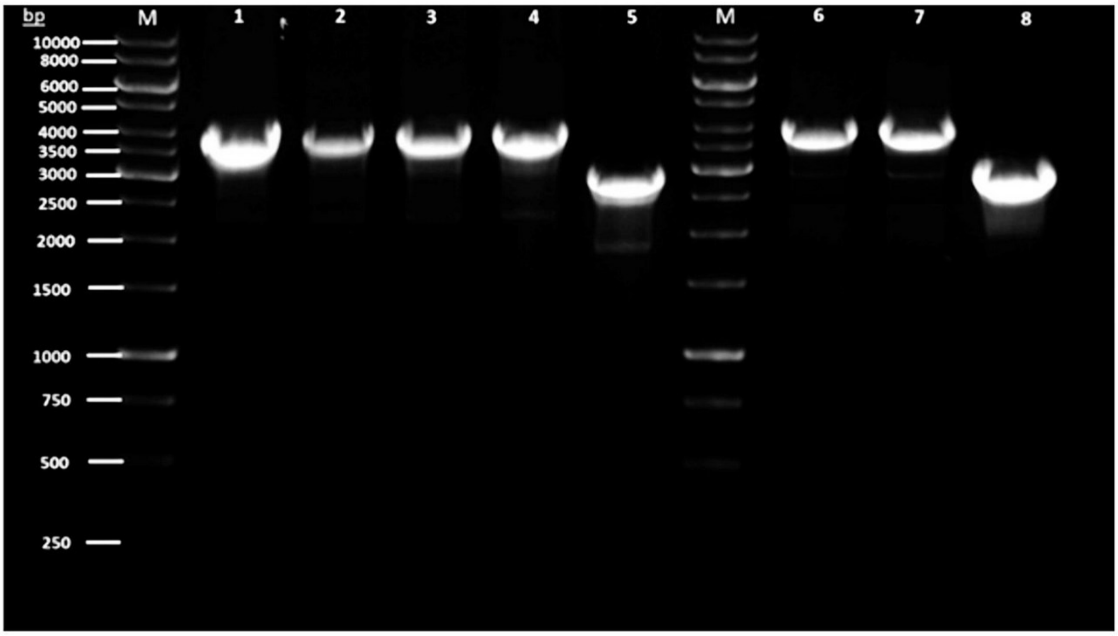

FIG S8 PCR analysis of the transformants. Agarose gel electrophoresis image of PCR products obtained with the nested primers McSVF1a_7-McSVF1a_8 for *svf1a* and McSVF1b_7-McSVF1b_8 for *svf1b*; M: GeneRuler 1 kb DNA ruler (Thermo Scientific); (1) MS12+*pyrG*-Δ*svf1a*/1 3848 bp; (2) MS12+*pyrG*-Δ*svf1a*/2 3848 bp; (3) MS12+*pyrG*-Δ*svf1a*/3 3848 bp; (4) MS12+*pyrG*-Δ*svf1a*/143848 bp; (5) *svf1a* in MS12 2926 bp; (6) MS12+*pyrG-Δsvf1b*/1 3873 bp; (7) MS12+*pyrG-Δsvf1b*/2 3873 bp; (8) *svf1b* in MS12 2684 bp.

TABLE S1 MICs of the different heavy metal agents against to *Mucor* strains

**Strains**	**MIC (µg/mL)**		
	$FeSO_4$	$MgSO_4$	$CuSO_4$
MS12+*pyrG*	2	>500	1.5
MS12+pyrG -*Δsvf1a*	2	>500	1
MS12+pyrG -*Δsvf1b*	2	>500	1.5

TABLE S2 Primers used in the present study

**primers**	**Sequence of primers 5'-3'**	**Amplified DNA**
	**Phusion PCR for *svf1a* deletion**	
McSVF1a_1	5'-ATAGTCGTTCCATCTATCGCTTGTC-3'	*svf1a* promoter and coding region upstream from protospacer
McSVF1a_2	5'-GACCATCTTTAGCTGTGTCACCT-3'	
McSVF1a_3	5'-AGACACCAGGTGACACAGCTAAA GATGGTCTGCCTCAGCATTGGTACTTG-3'	*pyrG* with own promoter
McSVF1a_4	5'-CAAACTTCTCGTCACCAACAGTAAT CTCGGGTACACTGGCCATGCTATCG-3'	
McSVF1a_5	5'-CCGAGATTACTGTTGGTGAC-3'	*svf1a* coding sequence downstream from protospacer and terminator seqences
McSVF1a_6	5'-CATCAAATGTCTAAGCTGCAC-3'	
McSVF1a_7	5'-CCTCTTCTGTGGAATCAAGCTC-3'	phusion pcr product
McSVF1a_8	5'-TCATAACCCTGAGGTCGTCTG-3'	
	**Phusion PCR for *svf1b* deletion**	
McSVF1b_1	5'-ATCTTGATGTCCTTCTCGTC-3'	*svf1b* promoter and coding region upstream from protospacer
McSVF1b_2	5'-CAGTGATGGTATCAGCTACAG-3'	
McSVF1b_3	5'-AGCATCAAGACTGTAGCTGATACC ATCACTGTGCCTCAGCATTGGTACTTG-3'	*pyrG* with own promoter
McSVF1b_4	5'-CAGCTTACGGATAACGTAGGGAAT CTCGGCGTACACTGGCCATGCTATCG-3'	
McSVF1b_5	5'-GCCGAGATTCCCTACGTTATCC-3'	*svf1b* coding sequence downstream from protospacer and terminator seqences
McSVF1b_6	5'-AGTTAGCCTTGTTGCTGAATCC-3'	
McSVF1b_7	5'GTAAATTCATCTTGGCGAGCAC-3'	phusion pcr product
McSVF1b_8	5'-CTAGCCATCGATCCTACCCTG-3'	
	**Primers used in the qRT-PCR experiments**	
McRTsvf1afw	5'-GTCGTTCACCTGTCGTTTCT-3'	*svf1a*
McRTsvf1arev	5'-CTTGGACAAGGTAGGGTCTAATG-3'	
McRTsvf1bfw	5'-GAGGACACCCAATACGACATTC-3'	*svf1b*
McRTsvf1brev	5'-CTCTGTTGTCATGGTGATGAGG-3'	
McRTactinfw	5'- CACTCCTTCACTACCACCGCTGA -3'	actin
McRTactinrev	5'- GAGAGCAGAGGATTGAGCAGCAG -3'	
MlLcb4afw	5'- CGACATTGATTCCTATGACACC -3'	*lcb4a*
MlLcb4arev	5'- CTGCCCTTGATGATTTGAAGTG -3'	
MlLcb4bfw	5'- ATGGATCTGTGCTCTGTGGT -3'	*lcb4b*

MlLcb4brev	5'- TGAATCTTGCTCTTGTTGGACTC -3'	
MlLcb4cfw	5'- TGTGTCCAAGTCAAGATACCT -3'	*lcb4c*
MlLcb4crev	5'- ACCATCAAATCCTCCATCGT -3'	
MlLcb3fw	5'- GAGTATTCAATCTTCCTCACCGT -3'	*lcb3*
MlLcb3rev	5'- CACTTCTTCCCTGACACCAC -3'	
MlYpkafw	5'- CTGTGGATTCAGCCTACGAC -3'	*ypka*
MlYpkarev	5'- GTGGTCCCAGTGTAACTCTC -3'	
MlYpkbfw	5'- AAACATGACTGATTCTGAGCGA -3'	ypkb
MlYpkbrev	5'- CATTTCATACAATAGCACGCCC -3'	
